# FGFR1/FOXM1 pathway: a key regulator of glioblastoma stem cells radioresistance and a prognosis biomarker

**DOI:** 10.18632/oncotarget.25827

**Published:** 2018-08-03

**Authors:** Valérie Gouazé-Andersson, Marie-Julie Ghérardi, Anthony Lemarié, Julia Gilhodes, Vincent Lubrano, Florent Arnauduc, Elizabeth Cohen-Jonathan Moyal, Christine Toulas

**Affiliations:** ^1^ Institut National de la Santé et de la Recherche Médicale (INSERM) UMR1037/Université Toulouse III Paul Sabatier, Cancer Research Center of Toulouse (CRCT), Toulouse, F-31000, France; ^2^ Institut Claudius Regaud, IUCT-O, Toulouse, F-31059, France; ^3^ CHU PURPAN-Pavillon Baudot, Place du Dr Baylac, Toulouse-Cedex 3, 31024, France

**Keywords:** FGFR1, FOX, glioblastoma, radioresistance, prognosis biomarker

## Abstract

Glioblastoma are known to be aggressive and therapy-resistant tumors, due to the presence of glioblastoma stem cells inside this heterogeneous tumor. We investigate here the involvement of FGFR1 in glioblastoma stem-like cells (GSLC) radioresistance mechanisms. We first demonstrated that the survival after irradiation was significantly diminished in FGFR1-silenced (FGFR1-) GSLC compared to control GSLC. The transcriptome analysis of GSLCs FGFR1(-) showed that FOX family members are differentially regulated by FGFR1 inhibition, particularly with an upregulation of FOXN3 and a downregulation of FOXM1. GSLC survival after irradiation was significantly increased after FOXN3 silencing and decreased after FOXM1 inhibition, showing opposite effects of FGFR1/FOX family members on cell response to ionizing radiation. Silencing FGFR1 or FOXM1 downregulated genes involved in mesenchymal transition such as GLI2, TWIST1, and ZEB1 in glioblastoma stem-like cells. It also dramatically reduced GSLC migration. Databases analysis confirmed that the combined expression of FGFR1/FOXM1/MELK/GLI2/ZEB1/TWIST1 is significantly associated with patients overall survival after chemo-radiotherapy treatment. All these results, associated with our previous conduced ones with differentiated cells, clearly established that FGFR1-FOXM1 dependent glioblastoma stem-like cells radioresistance pathway is a central actor of GBM treatment resistance and a key target to inhibit in the aim to increase the sensitivity of GBM to the radiotherapy.

## INTRODUCTION

Glioblastoma (GBM) is the most common and lethal primary brain tumor in adults. The standard treatment includes surgery followed by an association of radiotherapy with Temolozomide [1]. Almost all the patients will die of a relapse in radiation fields or away from the radiation fields, in the brain parenchyma. Our previous results have shown that factors controlling the microenvironment, such as basic fibroblast factor (FGF-2) [2, 3] induce a radioresistant phenotype [4]. Because FGF-2 binds to FGFR-1, 2 and 4 (for review [5]), we then examined the role of these receptors in GBM radioresistance. We first showed that FGFR1 in tumor cells is independent of bad prognostic factors of overall survival and time to progression in glioblastoma [6]. We recently showed that silencing FGFR1 induces an *in vitro* and *in vivo* radiosensitization of GBM cell lines via PLCγ and HIF1α [7]. These data led us to hypothesize that FGF2/FGFR1 pathway might be a central pathway sustaining the GBM cell radioresistance.

However, our view of the GBM treatment-resistance changed a decade ago by the discovery of the presence within the tumor of a subpopulation of self-renewing and pluripotent GBM stem cells (GSC), also called GBM initiating cells. These GSC are characterized by (i) their ability to self-renew *in vitro* (through the formation of neurospheres) and *in vivo* [8], their higher expression of neural stem cell markers (i.e. Olig2, Nestin or A2B5) and stem cell transcription factors (i.e. Sox2, Nanog, Gli1 or Oct4), (iii) their pluripotent aptitude to differentiate into neurons, astrocytes or oligodendrocytes and (iv) their high tumorigenic potential in orthotopically xenografted athymic nude mice [9]. In addition, the presence of these GSC may explain the high GBM recurrence rate, since this stem cells population was also shown to be highly tumorigenic and extremely radioresistant [10]. The treatment-resistance of these GBM stem cells has been largely investigated. Considering that FGFR1 regulates GBM differentiated cells radioresistance [7] and the primordial role of FGF2 in GSC maintenance, we investigate here whether FGFR1 may regulate glioblastoma stem-like cells (GSLC) radiosensitivity. We bring to light a new biological FGFR1 pathway sustaining GSLC radioresistance and show that the expression of these pathway effectors is predictive of the overall survival of GBM patients treated by chemo-radiotherapy.

## RESULTS

### FGFR1 inhibition increases glioblastoma stem-likecells sensitivity to ionizing radiation

**Figure 1:**
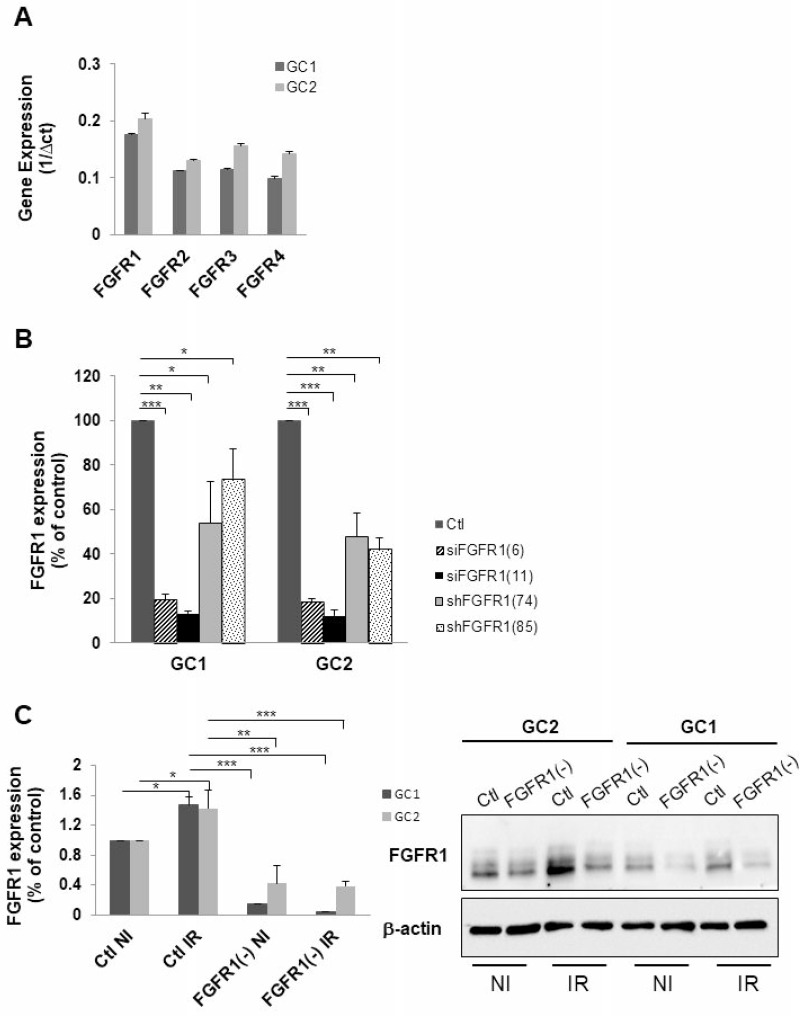
Down-regulation of FGFR1 gene expression in tumor cells derived from human GBM biopsy specimens (**A**) Expression of FGFR1, FGFR2, FGFR3 and FGFR4 was analyzed by real time PCR in tumor cells derived from 2 human GBM biopsy specimens (GC1 and GC2) cultured as GSLC-enriched neurospheres. (**B**) Cells were transfected with 2 different FGFR1 siRNA (siFGFR1(6) or siFGFR1(11)) or 2 different shRNA targeting FGFR1 (shFGFR1(74) or shFGFR1(85)) or a scramble control (Ctl). FGFR1 mRNA expression was analyzed by real-time PCR. (**C**) Cells were transfected with siFGFR1(11) (FGFR1(-)) or scramble control (Ctl). 24 h post-transfection cells are irradiated (6 Gy). 48 h post-irradiation FGFR1 expression was analyzed by real-time PCR and western-blot. Image is representative of 3 independent experiments. Quantifications of 3 experiments are presented as means ± SD. ^***^p < 0.001; ^**^p < 0.01; ^*^ 0.01 < *p* < 0.05.

**Figure 2:**
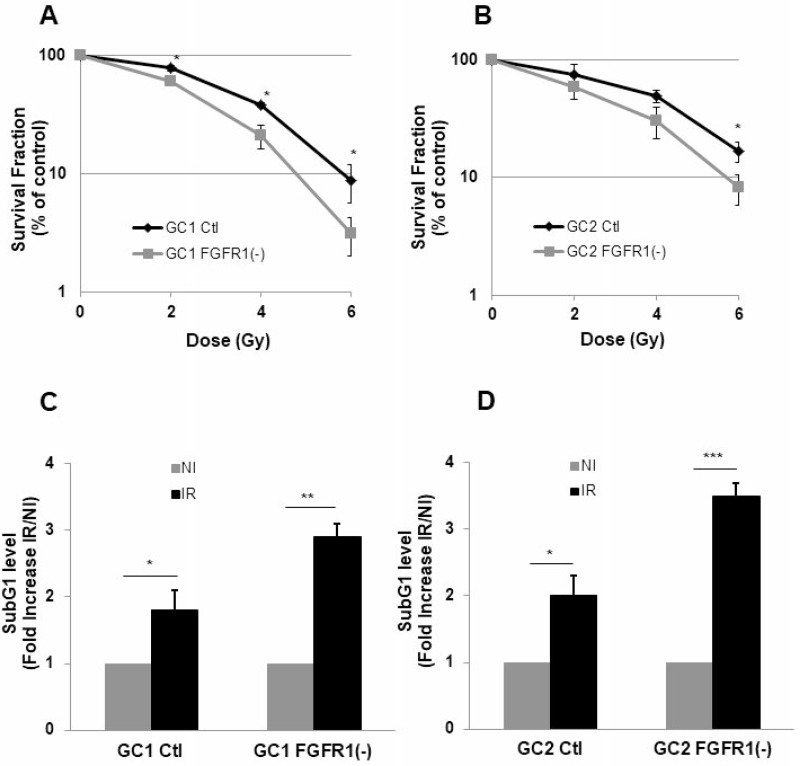
Down-regulation of FGFR1 gene expression radio-sensitizes and increases radio-induced cell death in tumor cells derived from GBM biopsy specimen Cells derived from 2 GBM biopsy specimen (GC1 and GC2) were transfected with siFGFR1(11) (GC1FGFR1(-) or GC2FGFR1(-)) or a scramble control (Ctl). (**A**–**B**) Cells were analyzed in clonogenic assay as described in “Materials and Methods”. (**C**–**D**) 48 h post-irradiation (4 Gy), propidium iodide staining was performed as described in “Materials and Methods” and the DNA content was analyzed by flow cytometry. Percentages of cells in sub-G1 are presented. Quantifications of 3 experiments are presented as means ± SD. ^***^p < 0.001; ^**^ *p* < 0.01; ^*^ 0.01 < *p* < 0.05.

Basal expression of FGFR receptors was examined in GSLC cell lines. GSLC showed similar level of basal FGFR, FGFR1 being the most expressed of the FGFRs receptors (Figure 1A). Inhibition of FGFR1 expression was obtained, as expected, by silencing FGFR1 with two different siRNA targeting FGFR1 and two shRNAs directed against FGFR1 (Figure 1B) without affecting their ability to form neurospheres (Supplementary Figure 1A and 1B). FGFR1 expression was similarly enhanced (1.5 times) 48 hours after a 4 Gy irradiation (Figure 1C). To investigate whether the specific inhibition of FGFR1 may modify the cellular radiosensitivity, we performed 3D clonogenic assay in the FGFR1 silenced GSLC cell lines. Surviving after irradiation was significantly diminished in FGFR1-silenced glioblastoma cells, GC1FGFR1(-) and GC2FGFR1(-), compared to control cells (Figure 2A and Figure 2B). To evaluate whether FGFR1 inhibition may activate radiation-induced cell death, we quantified subG1 fraction in a cytometry analysis. SubG1 level was increased in GC1FGFR1(-) and GC2FGFR1(-) compared to control cells by 61% and 75%, respectively (Figure 2C and Figure 2D) strongly suggesting that FGFR1 silencing increased glioblastoma stem-like cell death induced by radiation. These data showed that FGFR1 regulates GSLC radiosensitivity.

### Silencing of FGFR1 regulates FOX family members expression

To further investigate the biological pathways regulated by FGFR1 in GSLC, we then compared the transcriptomic profiles of GSLC silenced or not for FGFR1. As shown in Supplementary Figure 2, the pathways strongly downregulated in GCS2FGFR1(-) are implicated in cell cycle regulation and mitosis. A family of proteins involved in many cancers, known to regulate cell cycle, associated with stem cells and drug resistance (for review [11]) and whose expressions have been variously affected by FGFR1 silencing, has particularly attracted our attention, the Forkhead box (FOX) family. The analysis of GC2FGFR1(-) transcriptome showed that FOX family members are differentially regulated by FGFR1 inhibition (Table 1). Indeed FOXD3, FOXD4L3, FOXF2, FOXN3, FOXP2 are significantly upregulated whereas FOXI3, FOXM1, FOXRed1 are downregulated. These data strongly suggested that FOX family members might be regulators of FGFR1-induced radioresistance pathway in GSLC.

**Table 1:** FGFR1 inhibition induces modification in FOX family gene expression

Genes	Log2 Fold Change FGFR1(-) vs Ctl	Adjusted *p* value
FOXB1	0.993	0.815
FOXD3	1.117	0.04
**FOXD4**	**1.006**	**0.88**
FOXD4L1	1.021	0.73
**FOXD4L3**	**1.04**	**0.00425**
**FOXF2**	**1.172**	**0.00356**
**FOXI3**	**0.8496**	**0.0061**
FOXJ1	1.0678	0.2138
FOXK2	1.0183	0.2991
FOXL1	1.0646	0.47
FOXL2	1.0394	0.21
**FOXM1**	**0.8599**	**0.028**
**FOXN3**	**1.074**	**3.38E-06**
FOXN4	1.0043	0.9237
FOXP1	0.9669	0.253
**FOXP2**	**1.1573**	**0.0377**
FOXR2	1.0348	0.6368
**FOXRed1**	**0.8738**	**0.0035**

Cells derived from one GBM biopsy specimen (GC2) were transfected with siFGFR1(11) (GC2FGFR1(-)) or scramble control (Ctl). 48 h post-transfection, transcriptome analysis was performed as described in “Materials and Methods”.

### Opposite regulation of GBM stem-like cells radioresistance by FOX family members

**Figure 3:**
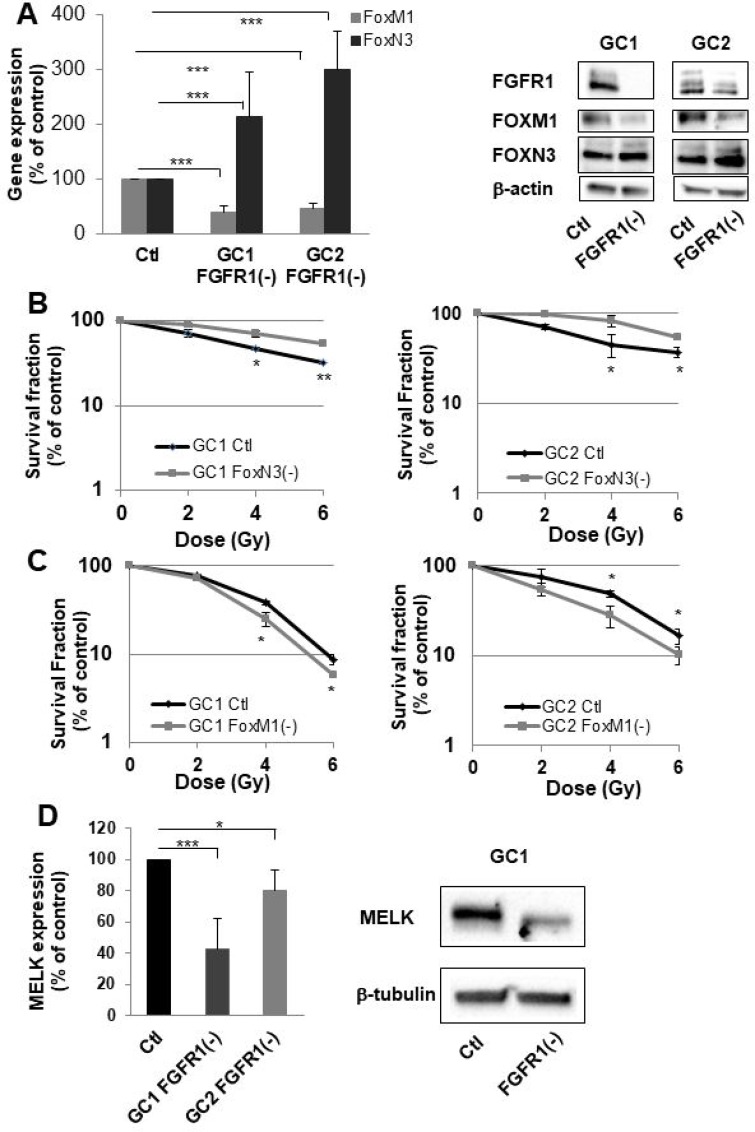
FGFR1 inhibition modifies FOXM1 and FOXN3 expression which are implicated in sensitization to radiation of cells derived from GBM biopsy specimen (**A**) Cells derived from 2 GBM biopsy specimen (GC1 and GC2) were transfected with siFGFR1(11) (GC1FGFR1(-) or GC2FGFR1(-)) or a scramble control (Ctl). 48 h post-transfection FOXM1 and FOXN3 expression was analyzed by real-time PCR and western-blot. Image is representative of 3 independent experiments. (**B**) Cells derived from 2 GBM biopsy specimen (GC1 and GC2) were transfected with siFOXN3 (GC1FOXN3(-) or GC2FOXN3(-)) or a scramble control (Ctl). Cells were analyzed in clonogenic assay as described in “Materials and Methods”. (**C**) Cells derived from 2 GBM biopsy specimen (GC1 and GC2) were transfected with siFOXM1 (GC1FOXM1(-) or GC2FOXM1(-)) or a scramble control (Ctl). Cells were analyzed in clonogenic assay as described in “Materials and Methods”. (**D**) 48 h post-transfection MELK expression was analyzed by real-time PCR and western-blot in GC1FGFR1(-), GC2FGFR1(-) and Ctl cells. Image is representative of 3 independent experiments. Quantifications of 3 experiments are presented as means ± SD. ^***^p < 0.001; ^**^p < 0.01; ^*^0.01 < *p* < 0.05.

To investigate the role of the FOX family in FGFR1-mediated radioresistance, we have chosen to specifically study two members whose expressions were the most affected by FGFR1 inhibition ie FOXN3 and FOXM1 (Table 1). We first checked by RT-PCR that silencing FGFR1 in GSLCs induced an overexpression of FOXN3 and a downregulation of FOXM1. As shown in Figure 3A (left panel), FOXN3 expression is significantly increased in GC1FGFR1(-) and GC2FGFR1(-) compared to control cells by 2.1 fold (*p* < 0.05) and 2.4 fold (*p* < 0.01), respectively, while FOXM1 is significantly decreased (2 fold in GSLC FGFR1(-) compared to GSLC control cells). The same result was obtained when analyzing FOX proteins (Figure 3A, right panel). We then performed clonogenic assay in FOXN3 or FOXM1 silenced GSLC to evaluate their respective roles in the control of intrinsic cellular radiosensitivity. The survival after irradiation of GSLC (GC1 and GC2) was significantly increased after FOXN3 silencing (Figure 3B) while FOXM1 inhibition significantly radiosensitizes GC1 and GC2 cells (Figure 3C). These data demonstrated that at least two members of the FOX family regulate GSLC radiosensitivity in an opposite manner, depending on their respective FGFR1-dependent regulation of expression.

In cancer cells, FOXM1 forms a protein complex with MELK [12]. MELK-regulated phosphorylation of FOXM1 transcriptional activity and induces the expression of various mitotic regulators such as survivin, Aurora B and CDC25B. Based on the evidence that FOXM1 directly interacts with MELK in glioblastoma stem-like cells, MELK may orchestrate the priming event of the complex signaling toward p53, VEGF, and Wnt/β-catenin in cancers including GBM [13]. To investigate whether MELK may be an effector of the FGFR1/FOXM1 pathway, we then analyzed MELK expression in GC1 and GC2 FGFR1(-) cells by qPCR and confirmed it by western blot. As shown in Figure 3D, MELK expression decreased significantly by 60 and 20% when FGFR1 was inhibited in GC1 and GC2 cells. This result shows that FGFR1/FOXM1 pathway is mediated by MELK.

### FGFR1 or FOXM1 knockdown reduces the expression of EMT associated genes

**Figure 4:**
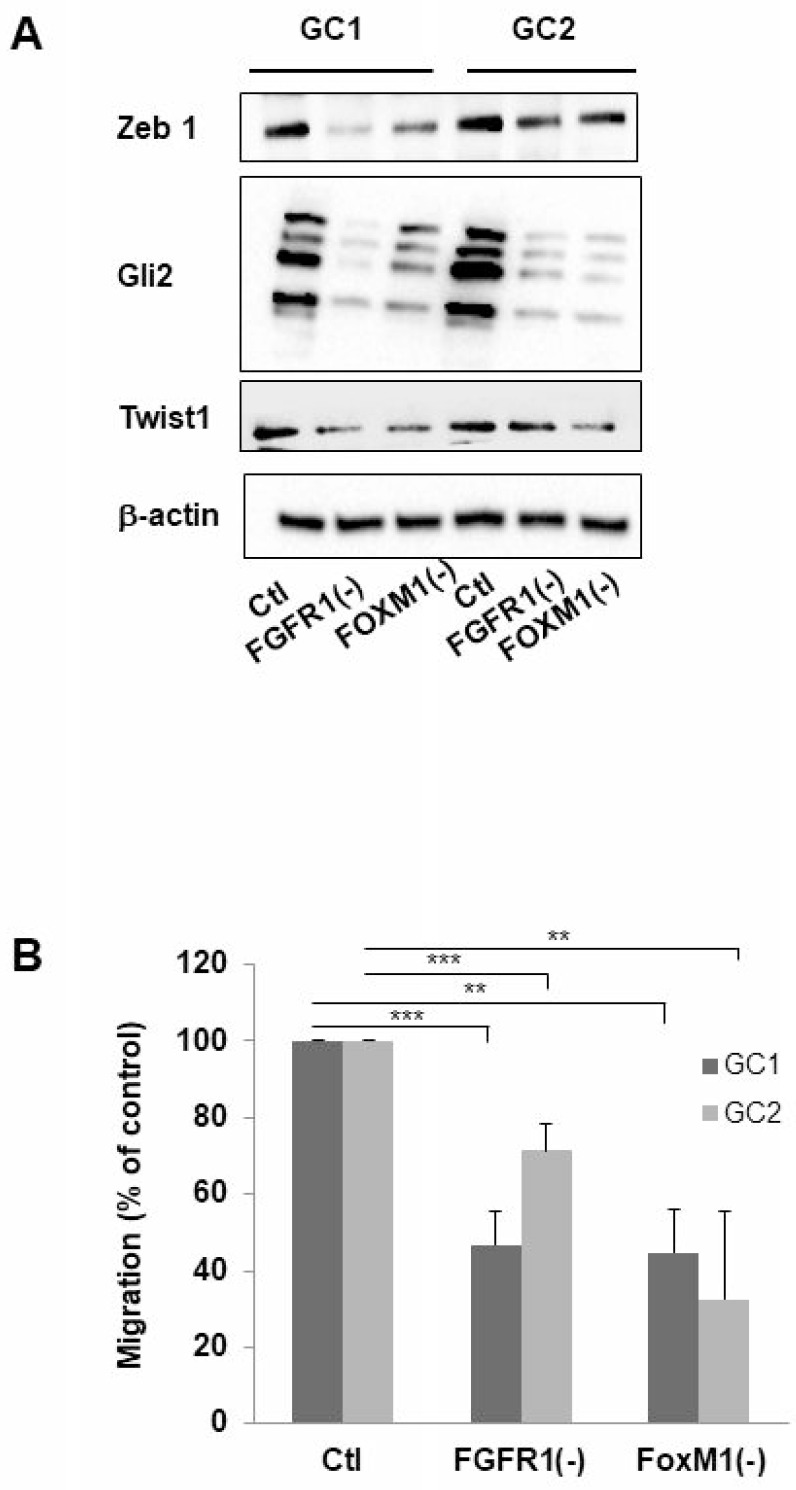
Inhibition of FGFR1 or FOXM1 modifies expression of genes implicated in mesenchymal transition and migration of cells derived from human GBM biopsy specimens Cells derived from 2 GBM biopsy specimen (GC1 and GC2) were transfected with siFGFR1(11) (GC1FGFR1(-) or GC2FGFR1(-)) or siFOXM1 (GC1FOXM1(-) or GC2FOXM1(-)) or a scramble control (Ctl). (**A**) 72 h post-transfection ZEB1, GLI2, TWIST1 expression was analyzed by western-blot in GC1FGFR1(-), GC1FOXM1(-), GC2FGFR1(-), GC2FOXM1(-) and Ctl. Image is representative of 3 independent experiments. β-actin was used as a loading control. (**B**) Cells were analyzed in migration assay as described in “Materials and Methods”. Quantifications of 3 experiments are presented as means ± SD. ^***^p < 0.001; ^**^p < 0.01; ^*^0.01 < *p* < 0.05.

The epithelial-to mesenchymal (EMT) or the glial-mesenchymal transition for brain tumors, process is increasingly recognized for playing a key role in the therapy resistance of tumors. During EMT, cells gain a migratory and invasive phenotype that is characteristic for mesenchymal cells; this phenotype has been recently linked to the resistance of the tumor to radiotherapy, particularly through FOXM1 a well-known actor of this mesenchymal transition [14]. To determine whether FGFR1 may be involved in the mesenchymal transition process, we performed western-blot targeting several proteins involved in EMT such as GLI2, ZEB1, and TWIST1 in GC1 or GC2FGFR1(-). In GC1 and GC2, a decrease of protein expression ZEB1, GLI2 and TWIST1 compared to control cells was observed when FGFR1 or FOXM1 was inhibited (Figure 4A). Furthermore, the analysis of GSLC migration in Boyden chambers revealed that GC1FGFR1(-) and GC1FOXM1(-) migrations were inhibited by 54% (29% for GC2) and 56% (68% for GSC2) respectively compared to control cells (Figure 4B) demonstrating that silencing FGFR1 or FOXM1 dramatically reduced GSC migration. Our results strongly suggested that FGFR1/FOXM1 pathway which regulate GSLC radioresistance may be a crucial actor of the mesenchymal transition.

### FGFR1/FOXM1/EMT genes is predictive of glioblastoma relapse for patients treated with chemo-radiotherapy protocol

**Figure 5:**
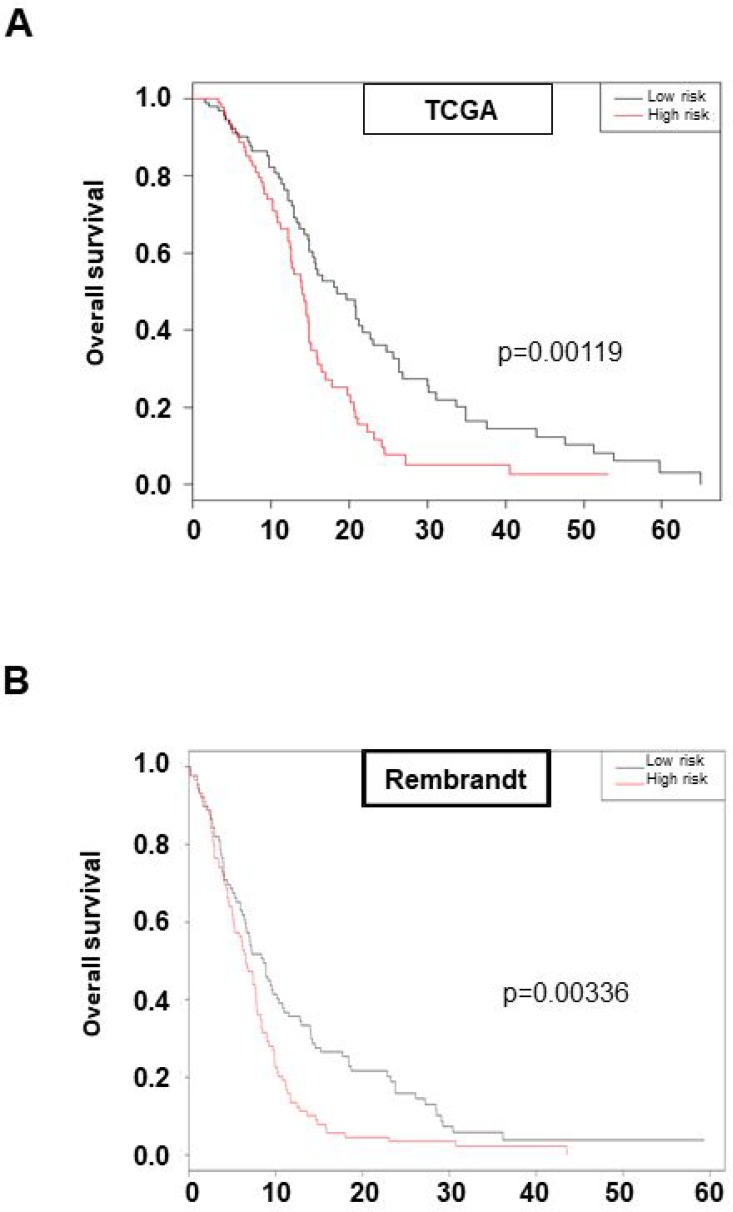
High expression of the six genes set: FGFR1/FOXM1/MELK/GLI2/ZEB1/TWIST1 is prognostic of survival of GBM patients Kaplan–Meier curves of overall survival in TCGA cohort (**A**) or Rembrandt cohort (**B**) stratified by six-gene prognostic set high and low risk. Statistical analysis was performed as described in “Materials and Methods”.

To determine whether this previously defined new pathway of GSLC radioresistance involving FGFR1, FOXM1, MELK, GLI2, TWIST1 and ZEB1 may be crucial for the *in vivo* GBM response to the standard treatment associating surgery and radio-chemotherapy, we investigated whether the associated expression of these six genes may be predictive of overall survival for patients treated with chemo-radiotherapy protocol. Using TCGA cohort (*n* = 184), we calculated a risk score from a Cox model including the six genes for each patient in the database and divide them into a high-risk group and a low-risk group by taking the mean value of risk score. Univariate analysis showed that our risk score and risk groups were significantly associated with overall survival (risk score: HR = 2.72 [1.66; 4.46], *p* = 7.7e-05 and High versus Low risk: HR = 1.85 [1.28; 2.68]; *p* = 0.00119 respectively) (Figure 5A). The median overall survival in the low-risk group was 18.4 months versus 14.0 months for the high-risk group (Figure 5A). Multivariate analysis showed that our six genes association remains a strong prognostic factor, independently of GBM common clinical and biological parameters as MGMT methylation status (HR = 2.82; *p* = 6.9e-05) (Table 2). Good prognostic ability was also found in the Rembrandt dataset (risk score: HR = 2.72 [1.69; 4.38]; *p* = 3.86e-05 and High versus Low risk: HR = 1.59 [1.17; 2.16]; *p* = 0.00336 respectively) (Figure 5B). These data led us to identify a six genes set defined from our *in vitro* results, involved in GSLC radioresistance and associated to patient overall survival when treated by the standard chemo-radiotherapy treatment and confirm our *in vitro* data demonstrating that FGFR1-dependant GSLC radioresistance pathways is a central actor of GBM treatment resistance.

**Table 2:** FGFR1/FOXM1/MELK/GLI2/ZEB1/TWIST1 set is a strong prognostic factor independently of clinical and biological parameters

	HR	*p*-value	95% Cl
Risk Score High vs Low	2.82	**6.9^e^-05**	[1.69–4.68]
Karno ≥ 70	0.82	0.521	[0.44–1.51]
Tumor resection vs others	1.70	0.070	[0.96–3.04]
Age ≥ 60	1.12	0.657	[0.68–1.84]
Mesenchymal vs Classical	1.17	0.617	[0.63–2.18]
Neural vs Classical	0.79	0.499	[0.40–1.56]
Proneural vs Classical	1.02	0.952	[0.47–2.24]
Non G-CIMP vs G-CIMP	2.23	0.129	[0.79–6.28]
MGMT methylated vs non methylated	0.54	**0.011**	[0.33–0.87]

## DISCUSSION

Our previous works have demonstrated that FGFR1 tumor expression was an independent prognostic factor of time to progression and overall survival in patients treated with radiotherapy for glioblastoma [6] and that inhibiting FGFR [15], and more specifically FGFR1 [7], increased the radiosensitivity of glioblastoma differentiated cells. To fully validate that this growth factor receptor may be a target to radiosensitize GBM, we have investigated here its role in the radiosensitivity of glioblastoma stem-like cells established from glioblastoma samples. FGF2/FGFR pathways have been largely reported to maintain cell stemness [16]. More specifically, FGFR1 has been described as governing self-renewal of adult neural stem cells [17]. If FGFR1 has already been involved in resistance to other therapy as hormone therapy or targeted drugs in different cancer models, little is known concerning its role in cancer stem cells response to therapy. Our present results clearly establish that silencing FGFR1 significantly increases GSLC sensitivity to ionizing radiation by increasing radiation-induced cell death.

In particular, our results bring to light the regulation of FOXM1 and FOXN3 belonging to the Forkhead box (FOX) family by FGFR1. The FOX family has been extended to include 44 members in humans which share a common DNA binding domain of up to 110 amino acids. All the FOX members are transcription factors but they have been shown to influence a diverse range of biological processes during development and throughout adult life. FOXM1, known for regulating the cell cycle via transcription of G1/S and G2/M transition factors, is upregulated in a multitude of cancer including glioblastoma. FOXM1 overexpression is associated with an increase in proliferation and tumorigenecity of cancer cells (for review [18]). A recent meta-analysis of several published studies revealed that elevated FOXM1 expression is associated with poor survival in most solid tumors [19]. FOXM1 expression has also been associated with resistance to chemotherapy [20] and to ionizing radiation of GBM cells [14, 21], unlike FOXN3 which inhibited growth, migration and invasion of colon cancer cells [22] and the proliferation of HCC cells [23]. In glioblastoma, low levels of FOXN3 mRNA expression were significantly associated with poor survival of patients not treated with chemotherapy or radiotherapy [24]. Our present data clearly showed that FOXN3 inhibition significantly decreased GSLC sensitivity to radiation *in vitro*. In this study, we established FOXN3 and FOXM1 regulation by a growth factor receptor. The relationship between growth factor receptor and FOXM1 has been reported for EGFR [25] but up to date, not for FGFR. In our model, the opposite regulations of FOXN3 and FOXM1 by FGFR1 result in adverse effect on GSLC radioresistance, strongly suggesting a differential role of FOX family members, at least two of them, in GBM radioresistance.

We then demonstrated that silencing FGFR1/FOXM1 pathway reduced the expression of EMT-associated genes as GLI2, ZEB1 and TWIST1 in GSLC. These three mesenchymal transition factors expressions have been linked to the tumor resistance to treatment: for example, ZEB1 promotes the resistance against temozolomide (TMZ), the standard-of-care chemotherapeutic today [26]. Knockdown of Gli2 restored sensitivity to vemurafenib-resistant melanoma cells [27] while TWIST1, which is overexpressed in colon cancer, plays a crucial role in the resistance of these tumors to irinotecan [28]. Other mesenchymal transition genes have been described in modulating glioma stem cells radioresistance [29]. Also, it has been recently shown that FGFR1 functions as a driver of EMT-associated drug resistance [30]. Our present results demonstrate that the FGFR1 pathway regulating GSLC radioresistance also affect GSLC migration *in vitro* strongly suggesting that FGFR1-dependent glial-mesenchymal transition may be linked to FGFR1-mediated GSLC cellular response to ionizing radiation through FOXM1, GLI2, ZEB1 and TWIST1.

To confirm the implication of FGFR1 pathway in GBM radioresistance *in vivo*, we raised the hypothesis that FOXM1, MELK, GLI2, TWIST1, ZEB1 could be involved in the worse survival prognosis of patients treated for GBM with chemo-radiotherapy. Our analysis of patients included in TCGA database confirmed that the combined expression of FGFR1/FOXM1/MELK/GLI2/ZEB1/TWIST1 was significantly associated with overall survival of patients treated with chemo-radiotherapy. The same conclusion has been drawn from Rembrandt database confirming that FGFR1-dependent pathway has a fundamental role in the GBM response to the treatment. Furthermore, these 6 genes set gathered three qualities: it was discovered from bench data obtained from GSLC established from patient samples; it involves a short list of genes; and it remains a solid prognostic factor, independently of GBM clinical or already known biological parameters. It could reasonably be hypothesized, even if complementally analyses should be performed, that this genes set might be useful to discriminate GBM patients whose tumor will respond to an association of a specific FGFR1 inhibitor with radiotherapy.

In conclusion, this work associated with our previous one [7], fully establishes that FGFR1 is a key target to inhibit in the aim to increase the sensitivity of GBM to the radiotherapy. Several clinical trials are evaluating the effect of FGFR inhibitors, the main part of these studies investigating the anti-tumoral effect of a TKI FGFR inhibitor alone on tumors presenting FGFR alterations ie amplifications or FGFR-fusion genes expression, most of them in association with a conventional drug. In particular, FGFR-TACC fusions which is clonal tumor-initiating events appear in 3% of glioblastoma for FGFR3- TACC3 fusion, confer strong sensitivity to FGFR tyrosine kinase in preclinical and preliminary clinical data [31]. Nevertheless, the frequency of fusion is considerably higher than the one of FGFR1-TACC1 rearrangements [32, 33]. Some of these trials investigate the FGFR inhibition as a means to overcome acquired resistance to various cancer treatments (for review [34]). Our results clearly showed that FGFR1 inhibition must be studied in association with radiotherapy and one would better investigate the potential radiosensitizer effect of FGFR1 inhibition than its anti-tumoral activity per se. In consequence, preclinical and then clinical trials should be designed to test the combination of drug specifically blocking FGFR1 with radiotherapy in the treatment of de novo glioblastoma.

## MATERIALS AND METHODS

### Human tumor collection

The study was conducted on newly diagnosed GBM tumor samples isolated from patients to establish primary GSLC cell lines (GC1 and GC2). These samples were all obtained after written informed consent from patients admitted to the Neurosurgery Department at Toulouse University Hospital and were processed in accordance with the Institution's Human Research Ethics Committee. Tumors used in this study were histologically diagnosed as grade IV astrocytoma according to the WHO criteria.

### Cell culture

The GBM samples were processed as described by Avril *et al.* [35] in order to obtain the corresponding primary neurospheres (NS) cell lines shown by other groups to be enriched in GSLC [35], NS GSLC lines were maintained in DMEM-F12 (Lonza, Levallois-Perret, France) supplemented with B27 and N2 (Invitrogen, Life Technologies, Saint Aubin, France), 25 ng/ml of FGF-2 and EGF (Peprotech, Neuilly sur Seine, France) at 37°C in 5% CO_2_ humidified incubators. All GSLC lines were used for the experiments in this medium between the second and twelfth passages, in order to avoid any stem cell characteristic loss.

To evaluate the role of FGFR1 in GSLC radiosensitivity, we established cultures of GSLCs (GC1 and GC2) from GBM samples (Supplementary Figure 3A). GSLCs expressed neural tumor stem cell markers NANOG, NESTIN, SHH, OLIG2, SOX2, NOTCH1, ITGA6, A2B5 and BIRC5 (Supplementary Figure 3B and 3C), were able to differentiate into neuronal-like and astrocytic-like cells and to express differentiation markers (GFAP, TUJ1, MAL, CTGF, and O4) [23] (Supplementary Figure 3C). Altogether, our data shows that GSLCs derived from our patient samples present GSLC characteristics.

### Targets silencing

Cells were transfected with different small interfering RNAs (siRNA): an aleatory sequence, SiScramble siRNA (5′-GACGUGGGACUGAAGGGGUdTdT3′), and two siRNAs specific for FGFR1, siFGFR1_6 (5′-CAGAGA TTTACCCATCGGGTA-3′) (QIAGEN, SI02224677, Courtaboeuf, France) and siFGFR1_11 (5′-CTGCATTG TGGAGAATGAGTA-3′) (QIAGEN, SI03094637, Courtaboeuf, France), against FOXM1, siFOXM1_8 (5′-G ACATTGGACCAGGTGTTTAA-3′), siFOXM1_7 (5′-TG GATCAAGATTATTAACCA-3′) (QIAGEN, SI04261831), against FOXN3 siFOXN3_1 (5′-CACGGCCAAATTAATT TACGA-3′) (Qiagen, SI00073647), siFOXN3_11 (5′-CTG CCTGACATCCGATTAGAA-3′) (Qiagen, SI03095029). Cells were transfected with 20 nM of the different siRNAs using the Lipofectamine RNAiMAX transfection reagent according to the manufacturer conditions (Invitrogen). For generating clones constitutively silencing FGFR1, cells have been transduced with a pool of 2 shRNAs directed against FGFR1; shFGFR1(74) (5′-TGCC ACCTGGAGCATCATAAT-3′) (Sigma-Aldrich, Mission Lentiviral Transduction Particles, CloneID: TRCN00003 12574) and shFGFR1(85) (5′-CCACAGAATTGGAGGC TACAA-3′) (Sigma-Aldrich, Mission Lentiviral Transduction Particles, CloneID: TRCN0000121185) or with an aleatory sequence, Scramble shRNA (ShScr) (Sigma-Aldrich, Mission Lentiviral Transduction Particles, CloneID: TRCN0000296111) according to the manufacturer recommendation (Sigma-Aldrich). Clones carrying shFGFR1 or shScr were selected and then maintained by continuously treating cells with G418 1 mg/μl.

### Quantitative real-time PCR

Total RNAs were isolated either from Neurospheres or GBM-differentiated cells using RNeasy kit (Qiagen, Hilden, Germany) and then reverse-transcribed using iScript cDNA synthesis kit (Bio-Rad, Hercules, CA, USA). Real-time qPCR reactions were carried out using Evagreen dye and ABI-Stepone+ Detection System (Applied Biosystems, Foster City, CA, USA) or the Fluidigm 96.96 dynamic array integrated fluidic circuits and the Biomark HD System according Advanced Development Protocol n°37 (Toulouse GeT Platform, France), β2-microglobulin (β2M) or Glyceraldehyde-3-phosphate dehydrogenase (GAPDH) was used as endogenous control in the ΔCt analysis. Amplification folds were measured by the 2^–ΔΔCt^ method. The different primers (Eurogentec, Liege, Belgium) used in this study were described in Supplementary Table 1.

### Flow cytometry analyses

Direct immunofluorescence assay was performed by FACS as previously described [35]. The antibodies used in this study were described in Supplementary Table 2. To evaluate the marker expression, we determined the specific fluorescence index (SFI) using the mean fluorescence intensity (MFI). The SFI was calculated with the following formula SFI = (MFI antibody - MFI isotype control) / MFI isotype control. The gating strategy used in these analyses is based on previously published protocol [35].

### Western blotting

Proteins were extracted with a buffer composed of 50 mM Tris-HCl pH = 7.5, 0.1% Triton X-100, EDTA 5 mM and a cocktail of proteases inhibitors. Proteins were separated on SDS-PAGE and then transferred onto a nitrocellulose membrane. Blots were probed with the following primary antibodies: anti-FGFR1 (D8E4) XP Rabbit (diluted 1:1000; #9740; Cell Signaling Technology), anti-FOXM1 (D12D5) XP Rabbit (diluted 1:1000; #5436; Cell Signaling Technology), anti-MELK (diluted 1:000; #2274, Cell Signaling Technology), anti-TCF8/ZEB1 (D80D3) Rabbit (diluted 1:000; #3396, Cell Signaling Technology), anti-GLI2 (C-10) (diluted 1:1000; #sc-271786, Santa Cruz biotechnology, Clinisciences), anti-TWIST1 (diluted at 2.5 μg/ml; LS-C30601, LSBio), anti-FOXN3/CHES1 (aa308-408) (diluted 1:1000; LS-C159675, LSBio), anti-Actin (1:20,000; Merk Millipore), anti-tubulin (diluted 1:000; #2146, Cell Signaling Technology). Detection was performed using peroxydase-conjugated secondary antibodies and chemilluminescence detection kit (ECL RevelBlot Plus and ECL RevelBlot Intense, Ozyme).

### Neurospheres formation/3D clonogenic assay

GC1 and GC2 neurospheres were dissociated and transfected with a control siRNA and siRNA targeting specifically FGFR1, FOXN3 or FOXM1. A day later, cells were seeded at a concentration of 100 cells/well in 96 well plates and irradiated at different doses (2, 4, and 6 Gy), using an Irradiator Gamma-cell Exactor 40 (Nordion, Ottawa, ON, Canada). After irradiation, survival cells constituted neurospheres. Neurospheres were counted when they are composed of at least 20 cells.

### Sub-G1 analysis

48 h post-irradiation, cells were fixed in 70% ice-cold ethanol for 1H at 4°C. After washing, the cell pellet was resuspended in propidium iodide (PI)-staining buffer (50 μg/ml PI, 10 μg/ml RNAse A) and incubated for 15 min at 37°C. The DNA content was analyzed by flow cytometry (BD AccuriTM C6 cytometer).

### Migration assay

GC1 and GC2 neurospheres were dissociated and transfected with a control siRNA and siRNA targeting specifically FGFR1 or FOXM1. Cell suspension (20,000 cells/chamber) was placed in upper chamber (Falcon, 8 μm pore size) priory coated with laminin (1 μg/ml). After 22 h, non-migratory cells were removed and migratory cells were fixed, stained with hematoxylin, and counted.

### RNA Isolation, quality assessment, probe preparation and GeneChip hybridization

Total RNA was isolated using an RNeasy Mini Kit (Qiagen), and eluted with nuclease-free water. All subsequent sample handling, labeling, and GeneChip (Human Gene 2.0 ST arrays, Affymetrix) processing was performed at the Genotoul Get laboratory (Toulouse, France; https://get.genotoul.fr/).

### Affymetrix analysis

The affymetrix chips were standardized by the RMA method (R software version 3.3.2, Bioconductor version 3.4). The differences between the different conditions were tested using an ANOVA (R software version 3.3.2) corrected for multiple tests using the Benjamini & Hochberg method. The gene lists were compared to different databases GeneOntology C5 [36], Reactome [37]) using the ZE Autocompare software based on an exact Zelen test [38]. All data are available at the National Center of Biotechnology Information (NCBI) GEO Database under the series number GSE116414.

### Statistical analysis

Student's test was performed to compare the means of values from different experiments. Differences were considered statistically significant at *p* < 0.05. Correlations between FGFR1 expression and other genes in the TCGA dataset were assessed using Spearman's rank correlation coefficient.

For survival analysis, using the glioblastoma database of TCGA (http://genome-cancer.ucsc.edu/), we focused on patients treated with standard chemo-radiotherapy for primary GBM, excluding patients with prior glioma history (*n* = 184 patients). Overall survival rates were estimated using Kaplan–Meier method and univariate analysis were performed using Cox proportional-hazards model or log-rank test.

Risk score was created from the linear predictor Xβ given by the multivariate Cox model including six genes (FGFR1/FOXM1/MELK/GLI2/ZEB1/TWIST1), where X is the expression matrix and β is the vector of coefficient estimates (Supplementary Table 3). Risk groups (poor versus good prognostic) were obtained by taking the median value of the risk score. A multivariate Cox regression analysis was used to adjust on standard clinical parameters. To confirm the prognostic ability of this gene set, we have fit a new Cox model on the Rembrandt dataset (*n* = 178) and built a new risk score for this cohort.

Two-sided *p*-values of less than 0.05 were considered statistically significant. Statistical analyses were performed using R 3.4.0 software.

## SUPPLEMENTARY MATERIALS FIGURES AND TABLES



## Abbreviations

EMT: Epithelial-to Mesenchymal Transition
FGF2: Fibroblast Growth Factor 2
FGFR: Fibroblast Growth Factor Receptor
GBM: Glioblastoma
GSLC: Glioblastoma Stem-like Cells
GSC: Glioblastoma Stem-Cells
IR: Irradiated
NI: Not Irradiated
NS: Neurosphere.
