# Supplementary material for: FGFR1/FOXM1 pathway: a key regulator of glioblastoma stem cells radioresistance and a prognosis biomarker
Source: Oncotarget. 2018 Aug 3;9(60):31637–49. doi: 10.18632/oncotarget.25827 (PMC6114977; doi:10.18632/oncotarget.25827)
Supplement: Supplementary file 1 [file oncotarget-09-31637-s001.pdf]

FGFR1/FOXM1 pathway: a key regulator of glioblastoma stem cells radioresistance and a prognosis biomarker

SUPPLEMENTARY MATERIALS

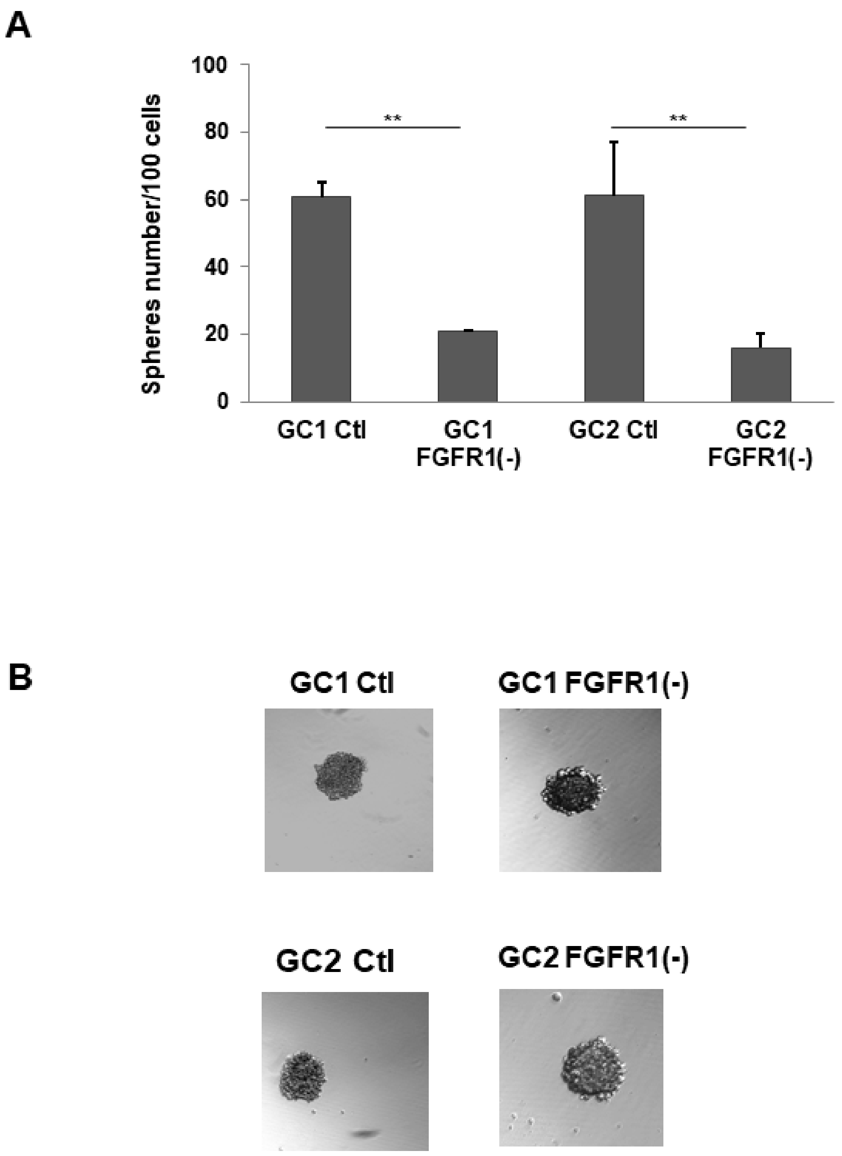

Supplementary Figure 1: Cells derived from human GBM biopsy specimens inhibited for FGFR1 form neurospheres. Cells derived from 2 GBM biopsy specimen (GC1 and GC2) were transfected with siFGFR1(11) (GC1FGFR1(-) or GC2FGFR1(-)) or a scramble control (Ctl). (**A**) Cells were analyzed for their capacity to form neurospheres as described in "Materials and Methods". Quantifications of 3 experiments are presented as means ± SD. $^{***}p < 0.001$; $^{**}p < 0.01$; $^{*}0.01 < p < 0.05$. (**B**) Phase contrast photomicrographs of GSLC-enriched neurosphere cell lines, magnification: ×10, scale bar: 6 µm.

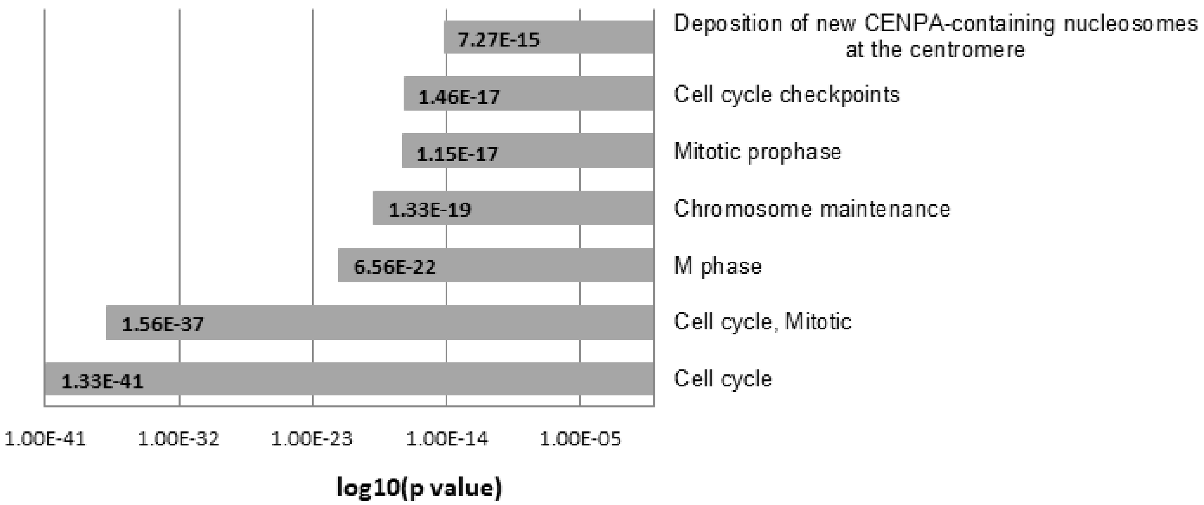

Supplementary Figure 2: FGFR1 inhibition induces a downregulation of cell cycle pathways. The genes list regulated in GCS2FGFR1(-) cells was compared to Reactome database according to "Materials and Methods".

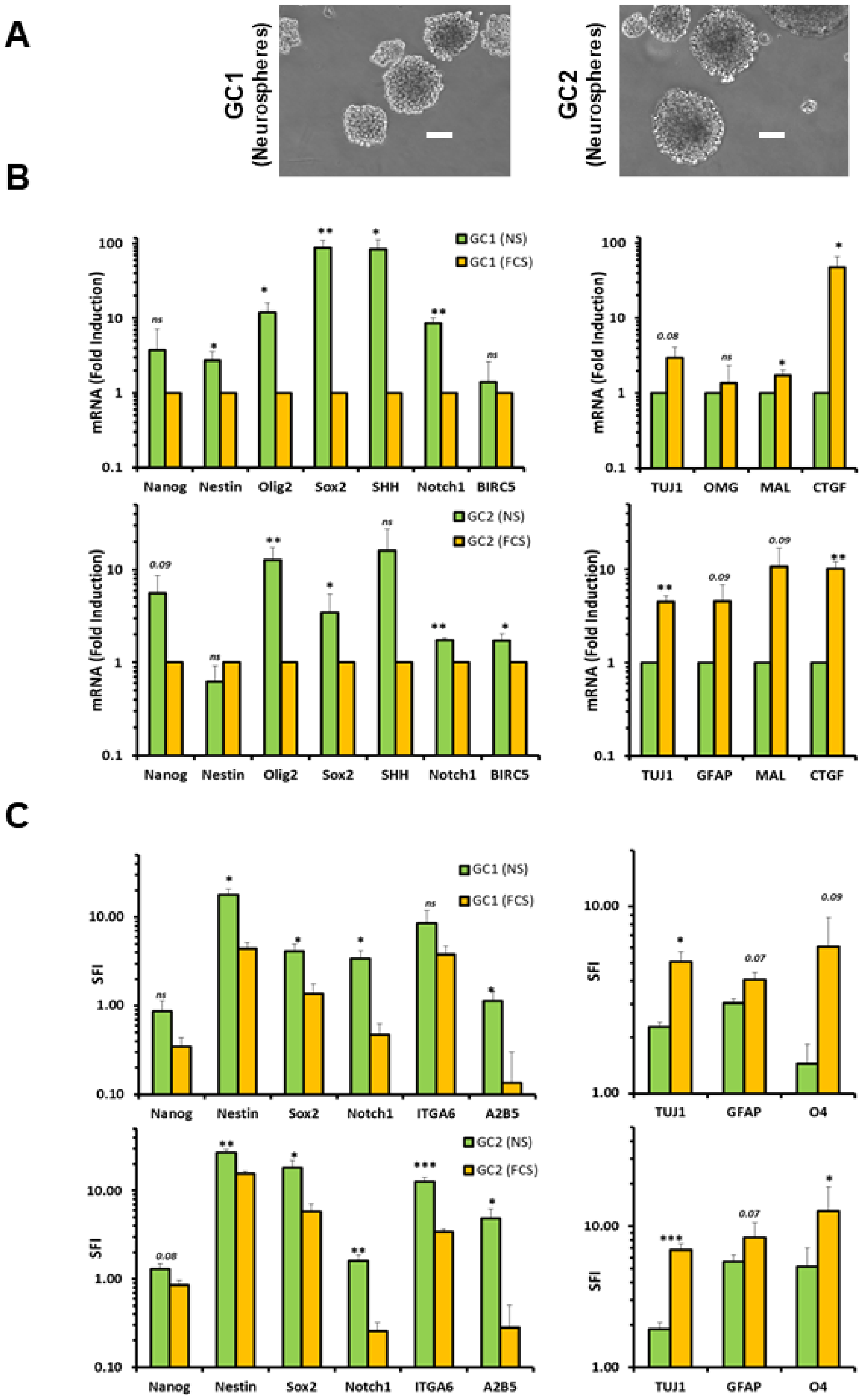

Supplementary Figure 3: Characterization of the stem and differentiated phenotypes in GSLC-enriched neurospheres and in differentiated GBM cultures (FCS). (**A**) Phase contrast photomicrographs of GSLC-enriched neurosphere cell lines isolated from 2 patient tumors (GC1, GC2). Magnification: ×10, scale bar: 6 µm. (**B–C**) GSLC-enriched neurosphere cell lines (GC1, GC2) were kept in stem cell medium or allowed to differentiate as adherent GBM cells for at least 15 days in FCS medium. (B) Real-time Quantitative PCR analysis of the stem (left panel) and differentiation (right panel) markers in neurospheres or GBM differentiated cells for both GC1 and GC2 cell lines. Shown are the fold inductions expressed as means ± SEM of at least 3 independent experiments. $^{*}p < 0.05$, $^{**}p < 0.01$ compared with the related control. ns: not significant. (C) Immunofluorescence FACS analysis of the stem (left panel) and differentiation (right panel) markers in neurospheres or GBM differentiated cells. The results, expressed as SFI (Specific Fluorescence Index), were representative of at least 3 independent experiments. $^{*}p < 0.05$, $^{**}p < 0.01$, $^{***}p < 0.001$ compared with the related control.

Supplementary Table 1: Forward and reverse primer sequences used for quantitative PCR detection

Gene (human)	Primer sequences	Type of marker
**β2-microglobulin**	forward 5′-ACCCCCACTGAAAAAGATGA-3′<br>reverse 5′-ATCTTCAAACCTCCATGATG-3′	endogenous control
**OMG**	forward 5′-TAGGGACTCCATGTTCTACCCA-3′<br>reverse 5′-TCTGCATCCCACTTACAGTGA-3′	Oligodendrocytic marker
**MAL**	forward 5′-CGCTGCCCTCTTTTACCTCAG-3′<br>reverse 5′-GAAGCCGTCTTGCATCGTGAT-3′	Oligodendrocytic marker
**GFAP**	forward 5′-GGCAAAAGCACCAAAGACGG-3′<br>reverse 5′-GGCGGCGTTCCATTTACAAT-3′	Astrocytic marker
**TUJ1**	forward 5′-GCTCAGGGGCCTTTGGACATCTCTT-3′<br>reverse 5′-TTTTCACACTCCTTCCGCACCACATC-3′	Neuronal marker
**CTGF**	forward 5′-CATCTCCACCCGGGTTACC-3′<br>reverse 5′-CAGGCGGCTCTGCTTCTCTA-3′	Differentiation marker
**Nanog**	forward 5′-GTCCCGGTCAAGAAACAGAA-3′<br>reverse 5′-TGCGTCACACCATTGCTATT-3′	Stemness-associated marker
**Nestin**	forward 5′-ATCGCTCAGGTCCTGGAAGG-3′<br>reverse 5′-AAGCTGAGGGAAGTCTTGGAG-3′	Stemness-associated marker
**Sox2**	forward 5′-GCACATGAACGGCTGGAGCAACG-3′<br>reverse 5′-TGCTGCGAGTAGGACATGCTGTAGG-3′	Stemness-associated marker
**Olig2**	forward 5′-CAGAAGCGCTGATGGTCATA-3′<br>reverse 5′-TCGGCAGTTTTGGGTTATTC-3′	Stemness-associated marker
**SonicHH**	forward 5′-GCGGAAGGTATGAAGGGAAG-3′<br>reverse 5′-GCCAAAGCGTTCAACTTGTC-3′	Stemness-associated marker
**Notch1**	forward 5′-TGGACCAGATTGGGGAGTTC-3′<br>reverse 5′-GCACACTCGTCTGTGTTGAC-3′	Stemness-associated marker
**BIRC5/Survivin**	forward 5′-CGAGGCTGGCTTCATCCA-3′<br>reverse 5′-AGAAGAAACACTGGGCCAAGTC-3′	Stemness-associated marker

Supplementary Table 2: Primary antibodies used for flow cytometry

Marker (human)	Antibodies and fluorochromes		Suppliers	FACS channel	Type of marker
**A2B5**	Mouse monoclonal antibody :<br>Isotype control :	A2B5 - APC<br>IgM - APC	Miltenyi	FL4-H	Stemness-associated marker
**ITGA6**	Rat monoclonal antibody :<br>Isotype control :	CD49f - PE<br>Igg2A -PE	eBioscience	FL2-H	Stemness-associated marker
**GFAP**	Mouse monoclonal antibody :<br>Isotype control :	GFAP - AF647<br>Igg2B - AF647	BD Biosciences	FL4-H	Astrocytic marker
**Nanog**	Goat polyclonal antibody :<br>Isotype control :	Nanog - PE<br>IgG - PE	R&D Systems	FL2-H	Stemness-associated marker
**Nestin**	Mouse monoclonal antibody :<br>Isotype control :	Nestin -Fluorescein<br>Igg1 - Fluorescein	R&D Systems	FL1-H	Stemness-associated marker
**Notch1**	Mouse monoclonal antibody :<br>Isotype control :	Notch1 - APC<br>Igg1 - APC	R&D Systems	FL4-H	Stemness-associated marker
**O4**	Mouse monoclonal antibody :<br>Isotype control :	O4 - PE<br>IgM - PE	Miltenyi	FL2-H	Oligodendrocytic marker
**TUJ1**	Mouse monoclonal antibody :<br>Isotype control :	Tuj1 - AF488<br>Igg2A - AF488	BD Biosciences	FL1-H	Neuronal marker
**Sox2**	Mouse monoclonal antibody :<br>Isotype control :	Sox2 - APC<br>Igg2A - APC	R&D Systems	FL4-H	Stemness-associated marker

Supplementary Table 3: Six-genes set and their corresponding coefficients

	**Coefficient**
**FGFR1**	0.27084
**FOXM1**	0.63132
**MELK**	−0.45751
**GLI2**	−0.60469
**TWIST1**	−0.00581
**ZEB1**	−0.01317

Coefficients determined as described in "Materials and Methods".
